# Paclitaxel-containing high-dose chemotherapy for relapsed or refractory testicular germ cell tumours

**DOI:** 10.1038/sj.bjc.6601664

**Published:** 2004-03-09

**Authors:** I A McNeish, E J Kanfer, R Haynes, C Giles, S J Harland, D Driver, G J S Rustin, E S Newlands, M J Seckl

**Affiliations:** 1 Department of Medical Oncology, Imperial College School of Medicine, Charing Cross Hospital, Fulham Palace Road, London W6 8RF, UK; 2 Department of Haematology, Imperial College School of Medicine, Hammersmith Hospital, London, UK; 3 Department of Medical Oncology, Meyerstein Institute of Oncology, Middlesex Hospital, London, UK; 4 Department of Medical Oncology, Mount Vernon Hospital, Harrow, Middlesex, England, UK

**Keywords:** Germ cell tumour, paclitaxel, high-dose chemotherapy, cisplatin resistance

## Abstract

High-dose regimes containing etoposide, carboplatin and an oxazaphospharine can salvage 30–40% of patients with relapsed or refractory male germ cell tumours (GCTs). The additional benefit of paclitaxel in such high-dose therapy has not been tested. Between March 1995 and November 2002, 36 male GCT patients were treated with Carbop-EC-T (paclitaxel 75 mg m^−2^, etoposide 450 mg m^−2^, carboplatin AUC 10 on days −7, −5 and −3 and cyclophosphamide 60 mg kg^−1^ on days −5 and −3) followed by peripheral blood stem cell infusion (day 0). The 1-year overall survival rate for all patients is 67% (median follow-up 29 months). For the 24 patients with cisplatin-sensitive disease, the 1-year overall and event-free survivals are 88 and 64%, respectively. For those with cisplatin refractory or absolutely refractory disease, the 1-year overall survival is 25%. In all, 12 patients relapsed at a median duration of 5 months, 11 of whom have died. There were also six treatment-related deaths, five associated with pneumonitis. Pulmonary toxicity has been reported with paclitaxel in other high-dose regimes. Since altering our protocol so that paclitaxel is infused over 24 h with steroid prophylaxis, only one of 18 patients (13 testicular GCTs and five other tumour types) has had a treatment-related death. Our results suggest that Carbop-EC-T may enable a greater proportion of patients with relapsed and refractory GCTs to enter long-term remission.

Germ cell tumours (GCTs) are extremely chemosensitive and up to 80% of patients with metastatic disease are cured with conventional cisplatin-based chemotherapy (Bower *et al*, 1997; Toner *et al*, 2001). However, for those patients with either relapsed, cisplatin-refractory disease or disease that is classified as IGCCCG poor prognosis (Mead *et al*, 1997), the outcome is much less favourable. For relapsed disease, conventional dose salvage regimes, such as VeIP (vinblastine, ifosfamide and cisplatin), can produce response rates of greater than 50%, but long-term cures are relatively rare (Loehrer *et al*, 1998). The first reports of the use of high-dose chemotherapy with peripheral blood stem cell support in heavily pretreated relapsed GCTs appeared in the late 1980s, showing long-term survival rates of 15–20% (Nichols *et al*, 1989). With improved patient selection and management, high-dose therapy with three drug regimes (usually etoposide, carboplatin and an oxazaphospharine) produced long-term survival rates of 30–40% (Beyer *et al*, 1996; Rick *et al*, 1999). Subsequently, high-dose treatment has been introduced increasingly early in the management of GCTs: as initial salvage, long-term survival rates of over 50% can be achieved (Bhatia *et al*, 2000) and as first-line treatment in those with poor prognosis disease, there is data from matched pair analysis to suggest a survival advantage for those treated with high-dose therapy compared to standard dose (Bokemeyer *et al*, 1999).

We have previously reported the outcome of 31 heavily pretreated patients who received high-dose carboplatin, etoposide and cyclophosphamide (Carbop-EC) followed by peripheral blood stem cell rescue (Lyttelton *et al*, 1998). There was one treatment-related death and the probability of event-free and overall survival at 3 years was 30 and 42%, respectively. In the patients with cisplatin-responsive relapses, overall survival was 60% at 3 years, but in patients with cisplatin-refractory disease, there were no survivors beyond 36 months. In an attempt to increase the rate of long-term survival, we introduced paclitaxel to this regime as it has activity in GCTs, both as a single agent (Motzer *et al*, 1994) and in combination (de Wit *et al*, 1999). Here, we report the outcome of the first 36 patients with relapsed or refractory testicular GCTs treated with this new regime, which we have named Carbop-EC-T.

## PATIENTS AND METHODS

### Patients

Between March 1995 and November 2002, we treated 36 patients with relapsed or refractory testicular GCTs. The details of the patients are listed in Table 1

**Table 1:** Patient characteristics

	**Carbop-EC-T**
	*N*=36
Median age	37.4 years (range 19–69)
	
Median follow-up	29 months (range 4–89)
	
Disease	
Teratoma	26
Seminoma	10
	
Median previous chemotherapy regimes	3 (2–6)
	
Sensitivity to cisplatin (Beyer's criteria)	
Sensitive	24
Refractory	7
Absolutely refractory	5
	
Prognostic risk score	
Good (score 0)	20
Intermediate (score 1–2)	12
Poor (score >2)	4
	
IGCCCG classification	
Good	26
Intermediate	5
Poor	5
	
Median estimated GFR	67 ml min^−1^ (range 30–146)
	
Left ventricular ejection fraction	58% (range 30–78%)
	
Lung function tests	
FEV1	87% predicted (range 47–122%)
*K*_CO_	96% predicted (range 68–142%)

. The protocol was subjected to the appropriate local institutional review and all patients gave written informed consent prior to treatment. The median age at the time of treatment was 37 years (range 19–69 years) and patients had received a median of 3 (range 2–6) cisplatin-containing chemotherapy regimes prior to high dose. The histology of the tumours was seminoma in 10 out of 36 (28%) and nonseminomatous germ cell tumour (NSGCT) in the remaining 26 patients (72%). At initial presentation, 26 out of 36 (72%) patients were classified as IGCCCG good prognosis, five out of 36 (14%) intermediate prognosis and five out of 36 (14%) poor prognosis. For seven out of 36 patients (19%), high-dose chemotherapy was given to intensify first salvage treatment, while 27 of the 36 (75%) had received more than one salvage regime prior to high-dose therapy. For the two remaining patients, high-dose therapy was given as intensification of first-line treatment after no response better than stable disease (SD) could be achieved with three consecutive standard dose regimes. Two patients had previously received Carbop-EC high-dose treatment and had relapsed after 12 and 24 months. Two patients received Carbop-EC-T twice; the first having SD after three standard dose regimes, but achieving a complete marker response to the first dose of Carbop-EC-T and so the second dose was given as consolidation. The second patient relapsed 6 months after the first Carbop-EC-T, but still had cisplatin-responsive disease and had sufficient peripheral blood stem cells stored to permit a second high-dose treatment.

By the criteria of Beyer *et al*. (1996) (see definitions below), 24 patients (67%) had cisplatin-sensitive disease, seven (19%) cisplatin-refractory disease and four (11%) were defined as absolutely refractory. Using the prognostic risk categories (Beyer *et al*, 1996) (see definitions below), 20 patients were good risk, 12 were intermediate and four were poor risk. In the majority of patients (33 out of 36), peripheral blood stem cells were harvested using a single bolus regime of high-dose etoposide (1.6 g m^−2^) followed by 12 daily injections of granulocyte colony-stimulating factor prior to leukapheresis (Kanfer *et al*, 1998). Prior to treatment, all patients had assessment of left ventricular function (trans-thoracic echocardiography and/or MUGA scanning), renal function (as measured by 24 h urinary creatinine clearance or EDTA clearance) and lung function (spirometry, lung volumes and gas transfer). The median estimated glomerular filtration rate was 67 ml min^−1^ (range 30–146). The median LV ejection fraction was 58% (range 30–78%), while the median forced expiratory volume in 1 s (FEV1) was 87% of predicted (range 47–122%) and the median carbon monoxide transfer (*K*_CO_) was 96% predicted (range 68–142%).

### Chemotherapy

The Carbop-EC-T regime is outlined in Table 2

**Table 2:** The Carbop-EC-T regime

*Day-7*	
Paclitaxel	75 mg m^−2^ over 3 h (now 24 h) with dexamethasone 20 mg 12 and 6 h prior
Etoposide	450 mg m^−2^ over 2 h
Carboplatin	AUC 10 (=10 × (GFR+25) mg) over 1 h
Dexamethasone	20 mg 12 and 6 h prior to paclitaxel

*Day-5*	
Paclitaxel	75 mg m^−2^ over 3 h (now 24 h) with dexamethasone 20 mg 12 and 6 h prior
Etoposide	450 mg m^−2^ over 2 h
Carboplatin	AUC 10 over 1 h
Cyclophosphamide	60 mg kg^−1^ over 1 h+MESNA 120 mg kg^−1^

*Day-3*	
Paclitaxel	75 mg m^−2^ over 3 h (now 24 h) with dexamethasone 20 mg 12 and 6 h prior
Etoposide	450 mg m^−2^ over 2 h
Carboplatin	AUC 10 over 1 h
Cyclophosphamide	60 mg kg^−1^ over 1 h+MESNA 120 mg kg^−1^

*Day 0*	
Reinfuse peripheral blood stem cells	2 × 10^6^ CD34+ve cells/kg

*Days 0–14 (last 13 patients only)*	
Prednisolone	30 mg b.d.

AUC=area under curve.

. Prior to receiving Carbop-EC-T, patients received conventional dose induction chemotherapy: 24 out of 36 patients received three cycles of TE/TP (paclitaxel 90 mg m^−2^ and etoposide 150 mg m^−2^ on day 1, paclitaxel 90 mg m^−2^ and cisplatin 60 mg m^−2^ on day 14, with treatment recommencing on day 29). Two patients, with EDTA clearances of under 40 ml min^−1^, received TE/TCarbo, in which cisplatin was substituted with carboplatin at a dose of area under curve (AUC) 5 mg ml min^−1^ according to the Calvert formula (dose=AUC × (GFR+25) mg) (Calvert *et al*, 1989). The remaining patients received a variety of induction regimes: POMB/ACE (Bower *et al*, 1997) (four patients), 5 day BEP (two patients), VeIP (two patients), EP (etoposide 500 mg m^−2^ and cisplatin 75 mg m^−2^ every 14 days: two patients) and gemcitabine (1 g m^−2^), carboplatin (AUC 5) paclitaxel (135 mg m^−2^: one patient).

Carbop-EC-T is a derivative of the regime that we have described previously (Lyttelton *et al*, 1998), itself a modification of a regime used at the Memorial Sloan-Kettering Cancer Center. It consists of etoposide 450 mg m^−2^ on days −7, −5 and −3 (total 1350 mg m^−2^), paclitaxel 75 mg m^−2^ on days −7, −5 and −3 (total 225 mg m^−2^), carboplatin AUC 10 on days −7, −5 and −3 (total dose AUC 30) and cyclophosphamide 60 mg kg^−1^ on days −5 and −3 (total dose 120 mg kg^−1^). Mesna was given to all patients to prevent urological toxicity. All patients received granisetron (3 mg i.v. per day), dexamethasone (4 mg b.d.) and domperidone (20 mg t.d.s.) on days −7 to 0 as antiemetic prophylaxis. In light of the observed toxicity (vide infra), the final 13 patients received each dose of paclitaxel over 24 h rather than 3 h with steroid prophylaxis of prednisolone 30 mg twice daily for 14 days commencing on day 0. All patients received 3 months of prophylactic antibiotics with ciprofloxacin, fluconazole and aciclovir. No additional prophylaxis was given to the 13 patients who received prednisolone. Pulmonary toxicity was treated with supportive care (oxygen and assisted ventilation as necessary).

### Definitions

Tumour sensitivity to cisplatin was classified according to the response to the last conventional dose induction chemotherapy regime prior to high-dose treatment. Tumours were defined as cisplatin sensitive if a response better than SD was achieved with no evidence of progression within 4 weeks. Cisplatin-refractory disease was defined when SD or better was achieved, but there was progression within 4 weeks, while absolutely refractory tumours progressed during the last cisplatin-based chemotherapy treatment (Rick *et al*, 1998).

In the prognostic scoring system (Beyer *et al*, 1996), having progressive disease (PD) prior to high-dose treatment, a mediastinal primary tumour and having refractory disease prior to high-dose treatment each gave a score of 1, while having absolutely refractory disease and an hCG value of >1000 IU l^−1^ prior to high-dose treatment each gave a score of 2. Patients who scored 0 overall were classified as good prognostic risk, those with score 1–2 were deemed intermediate risk and those with a score >2 were classified as poor risk. For the two patients who received Carbop-EC-T twice, the Beyer score was calculated at their first treatment.

### Statistical analyses

The probabilities of event-free and overall survival were calculated according to the technique of Kaplan and Meier (1958) and compared using log-rank analysis. All dates were calculated from day 0, the day of stem cell reinfusion. An event was defined as disease progression, disease relapse or death from any cause, which ever came first. Patients with no events were censored as of 1 November 2003. Statistical calculations were performed using Prism 3.0 software (GraphPad Software, San Diego, CA USA).

## RESULTS

**Figure 1:**
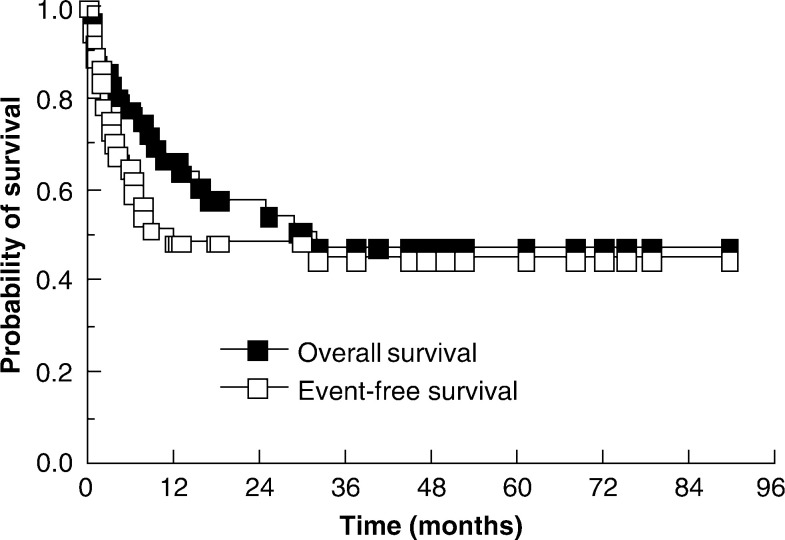
Estimates of event-free and overall survival for all 36 patients treated with Carbop-EC-T.

**Figure 2:**
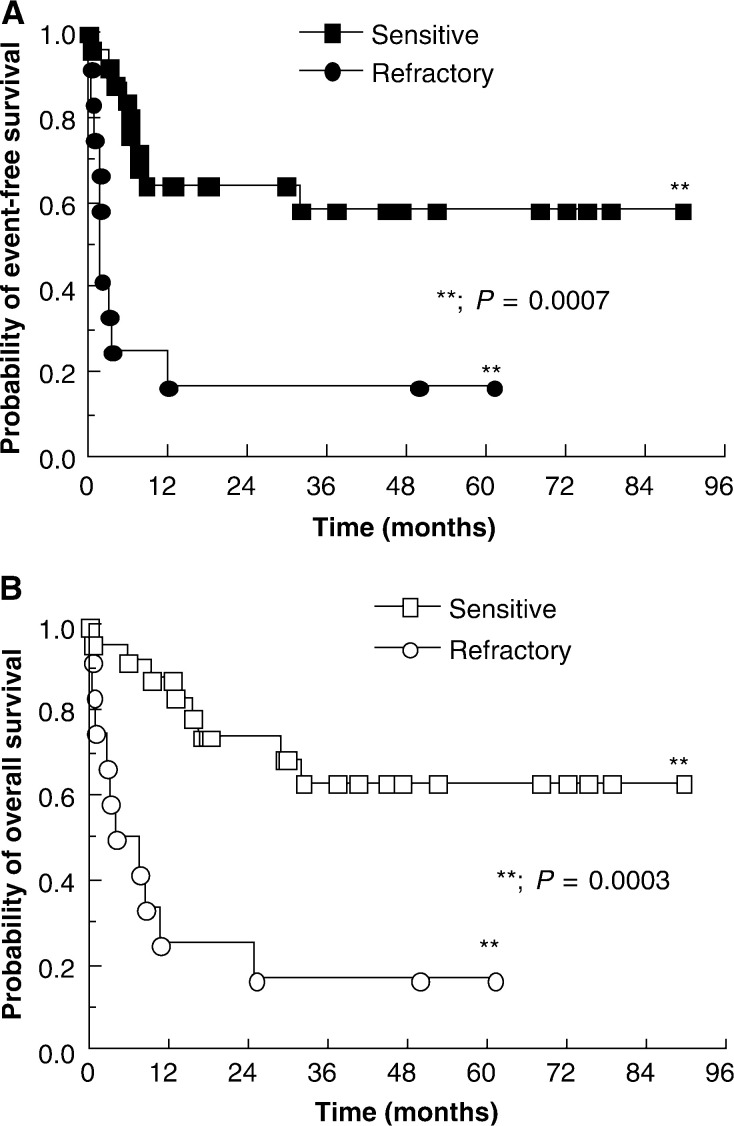
Estimates of event-free (**A**) and overall (**B**) survival for patients stratified according to cisplatin sensitivity by the criteria of Beyer *et al*.

With a median duration of follow- up of 29 months (range 4–89 months), the median event-free survival for all 36 patients is 12.1 months with a probability of event-free survival of 51 and 48% at 1 and 2 years, respectively. The median overall survival is 32.2 months and the probability of overall survival is 67 and 58% at 1 and 2 years, respectively (Figure 1

). As has been reported previously, patients with cisplatin-sensitive disease do considerably better than those with refractory disease and this is borne out in our data (Figure 2a and b

). Owing to the small numbers involved, patients with cisplatin-refractory and absolutely refractory disease have been analysed together. The median event-free and median overall survivals have not yet been reached for the 24 patients with cisplatin-sensitive disease, but the probability of event-free survival at both 12 and 24 months is 64% (Figure 2a) and, for overall survival, these probabilities are 88 and 74% (Figure 2b), respectively. By contrast, the median event-free and overall survival for the 12 patients with cisplatin-refractory disease is 2 months (comparison with cisplatin sensitive; *P*=0.0007) and 6 months (comparison with cisplatin sensitive; *P*=0.0003), respectively. However, two patients with refractory or absolutely refractory disease have survived without relapse for over 48 months. When analysed by tumour type, there is a nonsignificant trend for improved prognosis for patients with seminoma. The probability of overall survival at 24 months is 73% for the 10 patients with seminoma compared to 51% for the 26 patients with NSGCT, but the numbers are too small to permit firm conclusions from this comparison.

**Figure 3:**
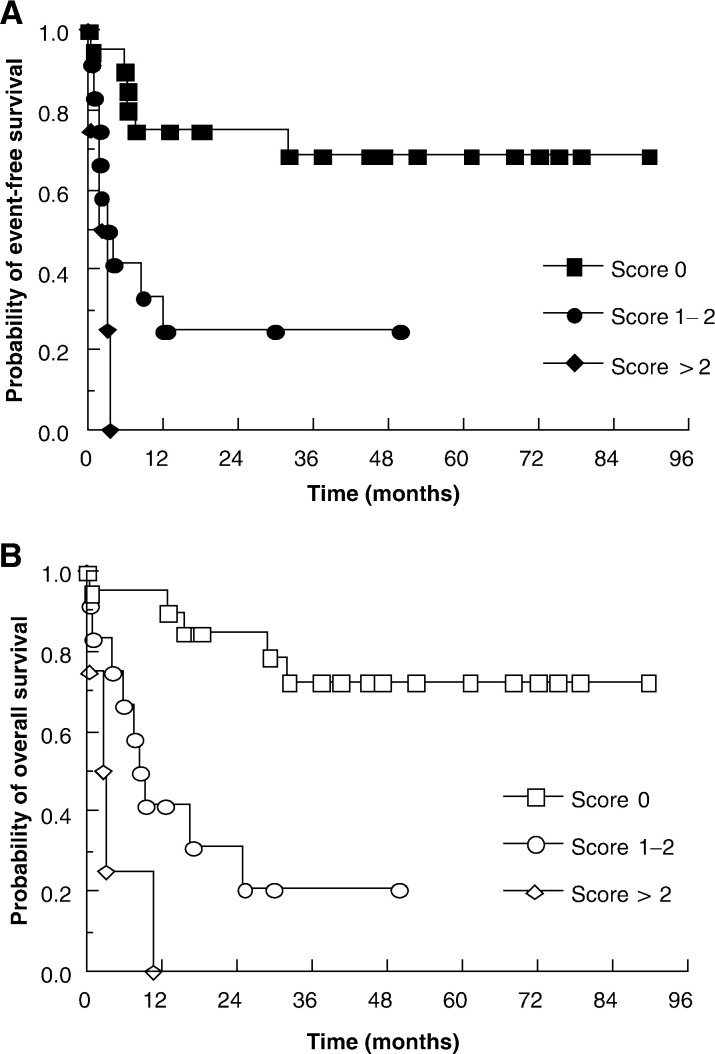
Estimates of event-free (**A**) and overall (**B**) survival for patients stratified according to the testicular GCT high-dose chemotherapy prognostic scoring system. Score 0, good prognosis; score 1–2, intermediate prognosis; score >2, poor prognosis.

We have also analysed patients according to the prognostic risk scores devised by Beyer *et al*. (1996) (Figure 3

). As expected, the patients who fall into the good prognostic group (score 0) have the best survival data; 12 month event-free survival 75%, 12 month overall survival 95%. However, even for those who fall into the intermediate group (score 1–2), outcomes are significantly worse: median event-free survival 3.5 months (comparison with good prognosis; *P*=0.004) and median overall survival 8.9 months (comparison with good prognosis; *P*=0.004). For the poor prognosis group, the outcomes are worse still: median event-free and overall survivals are both 2.6 months (comparison with good prognosis; *P*<0.0001 for both analyses) with no patient surviving for greater than 12 months.

Following Carbop-EC-T, 12 patients have relapsed at a median duration of 5 months (range 2–12), of whom 11 have subsequently died. Overall, there have been 18 deaths. In addition to the 11 patients with relapsed disease, one patient died of a secondary Non-Hodgkin's lymphoma that developed 31 months following Carbop-EC-T and which was refractory to treatment. There have been six treatment-related deaths (17%), of which five were associated with pneumonitis. Pulmonary toxicity has been reported to complicate other regimes that contain taxanes both at high dose (Damon *et al*, 1997) and at conventional doses (Dunsford *et al*, 1999; Wang *et al*, 2001). In an attempt to reduce this pulmonary toxicity, we altered the paclitaxel regime in December 2000 so that patients received each dose of paclitaxel as a 24 h infusion rather than over 3 h (Zimmerman *et al*, 1998) and also received prednisolone 30 mg twice daily for 2 weeks (Thomas *et al*, 2000). A total of 13 patients have now received Carbop-EC-T in this way and there has been one treatment-related death (8%). We have also used the 24 h paclitaxel Carbop-EC-T regime to treat two patients with relapsed ovarian GCTs and three patients with relapsed gestational choriocarcinoma, none of whom has had a treatment-related death. Thus, overall there has been only one treatment-related death in 18 patients (5.5%) treated with the modified regime.

The characteristics of the six patients who had treatment-related deaths showed no significant difference compared to the whole cohort for median age (37.4 *vs* 37 years), glomerular filtration rate (67 *vs* 75 ml min^−1^) or number of previous chemotherapy regimes (3 *vs* 3) (see Table 1). There was also no significant difference in the number of patients with IGCCCG poor or intermediate risk disease (three out of six compared to 10 out of 36; *P*=0.35). However, the patients who suffered treatment-related deaths were significantly more likely to have had cisplatin-refractory or absolutely refractory disease (five out of six compared to 12 out of 36; *P*=0.03) and to fall into the poor risk prognostic category (three out of six compared four out of 36; *P*=0.04). In light of the pulmonary toxicity of Carbop-EC-T, we wondered whether the presence of lung metastases or the prior effects of bleomycin increased the risk of developing pneumonitis. The incidence of lung metastases was no greater in patients who suffered treatment-related deaths than in the whole cohort, nor were there significant differences in lung function. Both the median FEV1 (66% predicted: range 47–87%) and K_C0_ (84% predicted; range 68–100%) were lower than those of the whole cohort, but the differences did not reach statistical significance.

We have previously treated 31 patients with relapsed or refractory testicular GSTs with the Carbop-EC regime that lacks paclitaxel but is otherwise the same as Carbop-EC-T (Lyttelton *et al*, 1998). The previous cohort of patients did not differ significantly from the current cohort in any of the characteristics detailed in Table 1 (See supplementary Table 3), and the addition of paclitaxel was not associated with any increase in nonpulmonary toxicity (including the rate of neutrophil recovery, the requirement for renal dialysis and the incidence of grade III/IV mucositis). Intriguingly, the two patients who had previously received Carbop-EC relapsing 12 and 24 months later and who were then retreated with Carbop-EC-T are currently both alive and well without relapse after 13 and 83 months, respectively. Taken together, these data suggest that Carbop-EC-T can salvage previous Carbop-EC chemotherapy failures and is probably no more toxic than Carbop-EC when the paclitaxel is administered as three 24 h infusions.

## DISCUSSION

Despite its widespread use, high-dose chemotherapy in the management of testicular GCTs has remained controversial for over a decade (Williams, 1992). The controversy has partially arisen because there have been no published randomised trials that compare high-dose and conventional dose treatments in relapsed or poor risk disease. The multicentre IT-94 trial, performed under the auspices of the European Group for Blood and Marrow Transplantation, has completed recruitment and an interim analysis has been presented in abstract form (Rosti *et al*, 2002). A total of 280 patients who relapsed after first-line platinum-containing chemotherapy were randomised either to four cycles of VIP/VeIP (cisplatin, ifosfamide and either etoposide or vinblastine) or three cycles of VIP/VeIP followed by high-dose Carbop-EC (carboplatin 1–2.2 g m^−2^ adjusted according to EDTA clearance, etoposide 1.8 g m^−2^, cyclophosphamide 6.4 g m^−2^). Of note, the study population differed in two respects from that presented here. Firstly, the IT-94 trial excluded patients classified as cisplatin refractory or absolutely refractory. Secondly, 23 patients with primary retroperitoneal disease and 24 with primary mediastinal disease were enrolled. At a median follow-up of 41 months, there was no difference between the study arms for 1-year event-free survival (approximately 50%) and the 3-year overall survival (53%). These results suggest that this nonpaclitaxel-containing type of high-dose schedule given to a population with cisplatin-sensitive disease is no better than standard-dose chemotherapy. The mature results of the IT-94 study have not yet been subjected to formal peer-review, but will be awaited with interest.

Direct comparisons between nonrandomised studies are illustrative, but fraught with difficulty, as there are inevitable differences in treatment regimes and patient parameters, especially in the extent of treatment prior to high-dose therapy. The first report of a large cohort was published in 1989 (Nichols *et al*, 1989), in which 33 heavily pretreated patients received etoposide (1200 mg m^−2^) and carboplatin (900–1200 mg m^−2^). Two-thirds of the patients had cisplatin-refractory disease and the treatment-related mortality was 21%. However, despite these adverse features, 44% of patients had objective responses and 10% survived over 1 year. Since then, there has been refinement both in terms of patient selection and supportive care. The largest single-centre study reported on 150 patients (Rick *et al*, 1998) treated with carboplatin (1500–2000 mg m^−2^), etoposide (1200–2400 mg m^−2^) and ifosfamide (up to 10 g m^−2^). In the final analysis of this cohort, two-thirds of patients had cisplatin-sensitive disease and over 60% fell into the good prognostic risk (score 0) group. Two-thirds of those treated received high-dose therapy as intensification of first salvage treatment and the median event-free and overall survivals were approximately 4 and 16 months, respectively. The predicted long-term event-free and overall survivals were 29 and 39%, respectively. Such figures have largely been reproduced by other groups (Siegert *et al*, 1994; Flechon *et al*, 1999; Nichols and Maziarz, 1999), including our own (Lyttelton *et al*, 1998). Where patients received high-dose chemotherapy exclusively as part of first salvage treatment, survival figures are improved, with nearly 60% of patients alive at a median follow-up of 39 months (Bhatia *et al*, 2000). However, interpretation of these last data is complicated by the fact that all patients received tandem high-dose therapy and 10 of the 65 patients were treated as part of a gene therapy protocol, with haematopoietic rescue consisting of *ex vivo* cytokine-stimulated, *mdr-1*-transduced CD34^+^ cells. Of 65 patients, 25, including the 10 gene therapy patients, also received maintenance oral etoposide.

In the absence of published randomised trial evidence, there has been one attempt to compare conventional and high-dose first salvage treatments in a matched pair analysis from two large patient databases (Beyer *et al*, 2002). The criteria were patients with proven relapsed NSGCT following first line cisplatin-based treatment who were then treated with cisplatin-based salvage treatment either with or without high-dose chemotherapy. In analyses of both overall and event-free survivals, there was a hazard ratio in statistically significant favour of high-dose treatment. However, at 2 years, the estimated absolute benefit in favour of high-dose chemotherapy was 6–12% for event-free survival and 9–11% for overall survival, which was smaller than was predicted from the single-centre phase I/II data.

Despite its activity in relapsed GCTs (Hinton *et al*, 2002; Motzer *et al*, 2000b), there have been no previous reports of high-dose chemotherapy that contains paclitaxel. There have been at least two reports of its use in the conditioning regime prior to high-dose therapy. In the first of these reports (Motzer *et al*, 2000a), two doses of paclitaxel (200 mg m^−2^) and ifosfamide (6 g m^−2^) followed by leukapheresis and three subsequent cycles of carboplatin (AUC 12–32) and etoposide (1200 mg m^−2^) with PBSC support were administered to 37 patients with cisplatin-resistant GCTs. Over 60% patients responded with 40% of these responses proving durable. In the second, 62 patients with relapsed or cisplatin-resistant GCTs received three cycles of paclitaxel (175 mg m^−2^), ifosfamide (6 g m^−2^) and cisplatin (100 mg m^−2^) followed by high-dose carboplatin (1500 mg m^−2^), etoposide (2400 mg m^−2^) and thiotepa (450–750 mg m^−2^) (Rick *et al*, 2001). In all, 66% of patients responded and, at 3 years follow-up, there was a 30% probability of overall survival and 25% probability of event-free survival.

When compared to these previous studies, the results presented here do suggest that the addition of paclitaxel to the high-dose regime can improve both event-free and overall survival. In a cohort in which the majority of patients had relapsed on more than one occasion and had received a median of three previous cisplatin-containing chemotherapy regimes, we estimate that, at 12 months, the probability of event-free survival is 51% and the probability of overall survival is high at 67%. At 2 years, these figures are 48 and 58%, respectively. To support further the notion that the addition of paclitaxel to high-dose therapy might improve the survival of poor risk GCT patients, we compared these results with our previously published series of patients who had received Carbop-EC alone. We appreciate that this is a retrospective analysis and not a randomised prospective trial and so chance and/or minor differences in patient prognostic variables could clearly have a major impact upon the results, but we believe that this comparison is meaningful since the two cohorts have been treated at the same centres and have very similar characteristics. Strikingly, this comparison revealed that the addition of paclitaxel to Carbop-EC generates a statistically significant improvement in event-free survival and may also improve overall survival (see supplementary Figure 1).

After treating 23 patients with Carbop-EC-T in which the paclitaxel was administered over 3 h, there had been five treatment-related deaths (22%), which compared to 3% in our previous series and 6% in the IT-94 trial. The majority of the deaths had been marked by pneumonitis. Taxane-induced pneumonitis was first described in 1995 (Goldberg and Vannice, 1995) and there have been many subsequent confirmatory reports, including in high-dose chemotherapy regimes (Khan *et al*, 1997; Prince *et al*, 2000). However, the cause remains uncertain, although postulated mechanisms have included delayed-type hypersensitivity and massive cytokine release (Fujimori *et al*, 1998). In patients with lung or breast cancers, previous irradiation may play a role (Khan *et al*, 1997), while the previous administration of bleomycin might potentially be relevant in our series. In the majority of reported cases, the taxanes were administered over 3 h. The responsiveness of the pneumonitis to corticosteroids varies greatly in published reports (Khan *et al*, 1997; Prince *et al*, 2000). However, there appears to be some evidence that the incidence of paclitaxel-induced pneumonitis can be reduced both by steroid prophylaxis (Thomas *et al*, 2000) and prolonged infusion of the drug (Zimmerman *et al*, 1998). Thus, we treated the subsequent 13 patients with prednisolone prophylaxis (30 mg b.d. for 14 days starting on day 0) and gave the paclitaxel as three 24 h infusions. We have also used this regime to treat two patients with relapsed ovarian GCTs and three patients with relapsed gestational choriocarcinoma. Only one of these 18 patients has suffered a treatment-related death (5.5%) suggesting that this altered Carbop-EC-T regime has reduced toxicity. Further patients will now need to be treated to confirm this.

Previous reports (Beyer *et al*, 1996; Lyttelton *et al*, 1998), including our current data, have shown that patients with cisplatin-refractory disease have significantly poorer outcomes than those who are cisplatin sensitive. However, it is noticeable that two patients presented here with cisplatin-refractory disease have survived long term (over 44 months), one of whom was absolutely refractory. Nevertheless, when patients are analysed according to the prognostic scoring system, those who fall into the poor risk group have a uniformly poor outcome as well as a very high probability of suffering a treatment-related death. Thus, we believe that careful consideration should be given to the appropriateness of administering high-dose therapy to those with the poorest prognosis.

In summary, we have described a paclitaxel-containing high-dose chemotherapy regime for relapsed or refractory GCTs that may significantly improve the survival rates in this disease when compared to other high-dose regimes. We believe that the extra toxicity of this regime can be minimised with combined steroid prophylaxis and prolonged paclitaxel infusion. Although these favourable results may simply reflect chance, we suggest that a paclitaxel-containing regime should be considered in any future randomised trial of high-dose chemotherapy in GCTs.

## References

[bib1] Beyer J, Kramar A, Mandranas R, Linkesch W, Greinix A, Croz J, Pico J, Diehl A, Bakemeyer C, Schmoll H, Nichols C, Einhorn L, Siegert W (1996) High-dose chemotherapy as salvage treatment in germ cell tumors: a multivariate analysis of prognostic variables. J Clin Oncol 14: 2638–2645 8874322 10.1200/JCO.1996.14.10.2638

[bib2] Beyer J, Stenning S, Gerl A, Fossa S, Siegert W (2002) High-dose *versus* conventional-dose chemotherapy as first-salvage treatment in patients with non-seminomatous germ-cell tumors: a matched-pair analysis. Ann Oncol 13: 599–605 12056711 10.1093/annonc/mdf112

[bib3] Bhatia S, Abonour R, Porcu P, Seshadri R, Nichols CR, Cornetta K, Einhorn LH (2000) High-dose chemotherapy as initial salvage chemotherapy in patients with relapsed testicular cancer. J Clin Oncol 18: 3346–3351 11013274 10.1200/JCO.2000.18.19.3346

[bib4] Bokemeyer C, Kollmannsberger C, Meisner C, Harstrick A, Beyer J, Metzner B, Hartmann J, Schmoll H-J, Einhorn L, Kanz L, Nichols C (1999) First-line high-dose chemotherapy compared with standard PEB/VIP chemotherapy in patients with advanced germ cell tumors: a multivariate and matched-pair analysis. J Clin Oncol 17: 3450–3456 10550141 10.1200/JCO.1999.17.11.3450

[bib5] Bower M, Newlands ES, Holden L, Rustin GJS, Begent RHJ (1997) Treatment of men with metastatic non-seminomatous germ cell tumours with cyclical POMB/ACE chemotherapy. Ann Oncol 8: 477–483 9233528 10.1023/a:1008279222625

[bib6] Calvert AH, Newell DR, Gumbrell LA, O'Reilly S, Burnell M, Boxall FE, Siddik ZH, Judson IR, Gore M, Wiltshaw E (1989) Carboplatin dosage: prospective evaluation of a simple formula based on renal function. J Clin Oncol 17: 1748–1756 10.1200/JCO.1989.7.11.1748 2681557

[bib7] Damon L, Rugo H, Wolf J, Breed C, Tripathy D, Ries C, Linker C (1997) Pulmonary injury complicates the addition of paclitaxel to high-dose alkylators and stem rescue in patients with advanced breast carcinoma. Blood 90: S1:4356

[bib8] de Wit R, Louwerens M, de Mulder PHM, Verweij J, Rodenhuis S, Schornagel J (1999) Management of intermediate-prognosis germ-cell cancer: results of a phase I/II study of Taxol-BEP. Int J Cancer 83: 831–833 10597204 10.1002/(sici)1097-0215(19991210)83:6<831::aid-ijc24>3.0.co;2-o

[bib9] Dunsford ML, Mead GM, Bateman AC, Cook T, Tung K (1999) Severe pulmonary toxicity in patients treated with a combination of docetaxel and gemcitabine for metastatic transitional cell carcinoma. Ann Oncol 10: 943–947 10509156 10.1023/a:1008377819875

[bib10] Flechon A, Biron P, Droz JP (1999) High-dose chemotherapy with hematopoietic stem-cell support in germ-cell tumor patient treatment: the French experience. Int J Cancer 83: 844–847 10597208 10.1002/(sici)1097-0215(19991210)83:6<844::aid-ijc28>3.0.co;2-6

[bib11] Fujimori K, Yokoyama A, Kurita Y, Uno K, Saijo N (1998) Paclitaxel-induced cell-mediated hypersensitivity pneumonitis – diagnosis using leukocyte migration test, bronchoalveolar lavage and transbronchial lung biopsy. Oncology 55: 340–344 9663424 10.1159/000011873

[bib12] Goldberg HL, Vannice SB (1995) Pneumonitis related to treatment with paclitaxel. J Clin Oncol 13: 534–535 7844618 10.1200/JCO.1995.13.2.534

[bib13] Hinton S, Catalano P, Einhorn LH, Loehrer PJ, Sr Kuzel T, Vaughn D, Wilding G (2002) Phase II Study of paclitaxel plus gemcitabine in refractory germ cell tumors (E9897): a trial of the Eastern Cooperative Oncology Group. J Clin Oncol 20: 1859–1863 11919245 10.1200/JCO.2002.07.158

[bib14] Kanfer EJ, McGuigan D, Samoson D, Abboudi Z, Abrahamson G, Apperley JF, Chilcott S, Craddock C, Davis J, MacDonald C, MacDonald D, Olavarria E, Philpott N, Rustin GJS, Seckl MJ, Sekhar M, Stern S, Newlands ES (1998) High-dose etoposide with granulocyte colony-stimulating factor for mobilization of peripheral blood progenitor cells: efficacy and toxicity at three dose levels. Br J Cancer 78: 928–932 9764585 10.1038/bjc.1998.603 PMC2063139

[bib15] Kaplan EL, Meier P (1958) Non-parametric estimation from incomplete observations. J Am Stat Assoc 53: 457–481

[bib16] Khan A, McNally D, Tutschka PJ, Bilgrami S (1997) Paclitaxel-induced acute bilateral pneumonitis. Ann Pharmacother 31: 1471–1474 9416383 10.1177/106002809703101205

[bib17] Loehrer PJ, Gonin R, Nichols CR, Weathers T, Einhorn LH (1998) Vinblastine plus ifosfamide plus cisplatin as initial salvage therapy in recurrent germ cell tumor. J Clin Oncol 16: 2500–2504 9667270 10.1200/JCO.1998.16.7.2500

[bib18] Lyttelton M, Newlands E, Giles C, Bower M, Guimaraes A, O'Reilly S, Rustin G, Samson D, Kanfer E (1998) High-dose therapy including carboplatin adjusted for renal function in patients with relapsed or refractory germ cell tumour: outcome and prognostic factors. Br J Cancer 77: 1672–1676 9635847 10.1038/bjc.1998.275 PMC2150058

[bib19] Mead GM, Stenning SP, Cook P, Fossa SD, Horwich A, Kaye SB, Oliver RTD, deMulder PHM, deWit R, Stoter G, Sylvester RJ, Bajorin DF, Bosl GJ, Mazumdar M, Nichols CR, Amato R, Pizzocaro G, Droz JP, Kramar A, Daugaard G, CortesFunes H, PazAres L, Levi JA, Colls BM, Harvey VJ, Coppin C (1997) International germ cell consensus classification: a prognostic factor-based staging system for metastatic germ cell cancers. J Clin Oncol 15: 594–6039053482

[bib20] Motzer RJ, Bajorin DF, Schwartz LH, Hutter HS, Bosl GJ, Scher HI, Lyn P, Fischer P (1994) Phase-II trial of paclitaxel shows antitumor-activity in patients with previously treated germ-cell tumors. J Clin Oncol 12: 2277–2283 7525885 10.1200/JCO.1994.12.11.2277

[bib21] Motzer RJ, Mazumdar M, Sheinfeld J, Bajorin DF, Macapinlac HA, Bains M, Reich L, Flombaum C, Mariani T, Tong WP, Bosl GJ (2000a) Sequential dose-intensive paclitaxel, ifosfamide, carboplatin, and etoposide salvage therapy for germ cell tumor patients. J Clin Oncol 18: 1173–1180 10715285 10.1200/JCO.2000.18.6.1173

[bib22] Motzer RJ, Sheinfeld J, Mazumdar M, Bains M, Mariani T, Bacik J, Bajorin D, Bosl GJ (2000b) Paclitaxel, ifosfamide, and cisplatin second-line therapy for patients with relapsed testicular germ cell cancer. J Clin Oncol 18: 2413–2418 10856101 10.1200/JCO.2000.18.12.2413

[bib23] Nichols C, Maziarz R (1999) High dose chemotherapy – results of American studies. Int J Cancer 83: 841–843 10597207 10.1002/(sici)1097-0215(19991210)83:6<841::aid-ijc27>3.0.co;2-c

[bib24] Nichols CR, Tricot G, Williams SD, Vanbesien K, Loehrer PJ, Roth BJ, Akard L, Hoffman R, Goulet R, Wolff SN, Giannone L, Greer J, Einhorn LH, Jansen J (1989) Dose-intensive chemotherapy in refractory germ-cell cancer – a phase-I/II trial of high-dose carboplatin and etoposide with autologous bone-marrow transplantation. J. Clin Oncol 7: 932–939 2544687 10.1200/JCO.1989.7.7.932

[bib25] Prince HM, Rischin D, Toner GC, Seymour JF, Blakey D, Gates P, Eerhard S, Chapple P, Quinn M, Brettell M, Juneja S, Wolf M, Januszewicz EH, Richardson G, Scarlett J, Briggs P (2000) Repetitive high-dose therapy with cyclophosphamide, thiotepa and docetaxel with peripheral blood progenitor cell and filgrastim support for metastatic and locally advanced breast cancer: results of a phase I study. Bone Marrow Transplant 26: 955–961 11100274 10.1038/sj.bmt.1702650

[bib26] Rick O, Beyer J, Kingreen D, Schwella N, Krusch A, Schlecher J, Kirsch A, Huhn D, Siegert W (1998) High-dose chemotherapy in germ cell tumours: a large single centre experience. Eur J Cancer 34: 1883–1888 10023310 10.1016/s0959-8049(98)00272-x

[bib27] Rick O, Bokemeyer C, Beyer J, Hartmann JT, Schwella N, Kingreen D, Neureither S, Metzner B, Casper J, Wandt H, Hartmann F, Schmoll HJ, Derigs G, Gerl A, Berdel WE, Kanz L, Siegert W (2001) Salvage treatment with paclitaxel, ifosfamide and cisplatin plus high-dose carboplatin, etoposide and thiotepa followed by autologous stem-cell rescue in patients with relapsed or refractory germ cell cancer. J Clin Oncol 19: 81–88 11134198 10.1200/JCO.2001.19.1.81

[bib28] Rick O, Siegert W, Beyer J (1999) High-dose salvage chemotherapy. Germ-cell tumor treatment results in Germany. Int J Cancer 83: 839–840 10597206 10.1002/(sici)1097-0215(19991210)83:6<839::aid-ijc26>3.0.co;2-a

[bib29] Rosti G, Pico J-L, Wandt H, Koza V, Salvioni R, Theodore C, Lelli G, Siegert W, Horwich A, Marangolo M, Schmoll H-J, Linkesch W, Pizzocaro G, Bouzy J, Kramar A, Droz J-P, Biron P (2002) High-dose chemotherapy (HDC) in the salvage treatment of patients failing first-line platinum chemotherapy for advanced germ cell tumors (GCT); first results of a prospective randomised trial of the European Group for Blood and Marrow Transplantation (EBMT): IT-94 study. Proc ASCO 21: 716 10.1093/annonc/mdi228 15928070

[bib30] Siegert W, Beyer J, Strohscheer I, Baurmann H, Oettle H, Zingsem J, Zimmermann R, Bokemeyer C, Schmoll H, Huhn D (1994) High-dose treatment with carboplatin, etoposide, and ifosfamide followed by autologous stem-cell transplantation in relapsed or refractory germ cell cancer: a phase I/II study. The German Testicular Cancer Cooperative Study Group. J Clin Oncol 12: 1223–1231 7911158 10.1200/JCO.1994.12.6.1223

[bib31] Thomas AL, Cox G, Sharma RA, Steward WP, Shields F, Jeyapalan K, Muller S, O'Byrne KJ (2000) Gemcitabine and paclitaxel associated pneumonitis in non-small cell lung cancer: report of a phase I/II dose-escalating study. Eur J Cancer 36: 2329–2334 11094306 10.1016/s0959-8049(00)00341-5

[bib32] Toner GC, Stockler MR, Boyer MJ, Jones M, Thomson DB, Harvey VJ, Olver IN, Dhillon H, McMullen A, Gebski VJ, Levi JA, Simes RJ (2001) Comparison of two standard chemotherapy regimens for good-prognosis germ-cell tumours: a randomised trial. Australian and New Zealand Germ Cell Trial Group. Lancet 357: 739–745 11253966 10.1016/s0140-6736(00)04165-9

[bib33] Wang GS, Yang KY, Perng RP (2001) Life-threatening hypersensitivity pneumonitis induced by docetaxel (taxotere). Br J Cancer 85: 1247–1250 11720456 10.1054/bjoc.2001.2071 PMC2375237

[bib34] Williams SD (1992) High dose therapy in germ cell tumors: when, what, and how much? Ann Oncol 3: 780–781 1337463 10.1093/oxfordjournals.annonc.a058095

[bib35] Zimmerman T, Grinblatt D, Malloy R, Williams S (1998) A phase I dose escalation trial of continuous infusion paclitaxel to augment high dose cyclophosphamide and thiotepa plus stem cell rescue for the treatment of patients with advanced breast carcinoma. Cancer 83: 1540–1545 9781947

