# Supplementary material for: Paclitaxel-containing high-dose chemotherapy for relapsed or refractory testicular germ cell tumours
Source: Br J Cancer. 2004 Mar 9;90(6):1169–75. doi: 10.1038/sj.bjc.6601664 (PMC2410221; doi:10.1038/sj.bjc.6601664)
Supplement: Supplementary File 1 [file 90-6601664x1.doc]

**Time (months)**

**Probability of event-free survival**

*

*

*; p = 0.033

**Time (months)**

**Probability of overall survival**

A

B

McNeish et al: Supplementary figure
